# Opposing role of condensin hinge against replication protein A in mitosis and interphase through promoting DNA annealing

**DOI:** 10.1098/rsob.110023

**Published:** 2011-12

**Authors:** Yuko Akai, Yumiko Kurokawa, Norihiko Nakazawa, Yuko Tonami-Murakami, Yuki Suzuki, Shige H. Yoshimura, Hiroshi Iwasaki, Yoshiharu Shiroiwa, Takahiro Nakamura, Eri Shibata, Mitsuhiro Yanagida

**Affiliations:** 1 Okinawa Institute of Science and Technology Graduate University, Onna-son, Okinawa 904-0495, Japan; 2 National Institute of Genetics, Mishima, Shizuoka 411-8540, Japan; 3 Graduate School of Biostudies, Kyoto University, Sakyo-ku, Kyoto 606-8501, Japan; 4 Tokyo Institute of Technology, Graduate School of Bioscience and Biotechnology, Nagatsuda, Yokohama 226-8501, Japan

**Keywords:** structural maintenance of chromosomes, DNA damage, mitosis, DNA metabolism, condensation

## Abstract

Condensin is required for chromosome dynamics and diverse DNA metabolism. How condensin works, however, is not well understood. Condensin contains two structural maintenance of chromosomes (SMC) subunits with the terminal globular domains connected to coiled-coil that is interrupted by the central hinge. Heterotrimeric non-SMC subunits regulate SMC. We identified a novel fission yeast SMC hinge mutant, *cut14-Y1*, which displayed defects in DNA damage repair and chromosome segregation. It contains an amino acid substitution at a conserved hinge residue of Cut14/SMC2, resulting in diminished DNA binding and annealing. A replication protein A mutant, *ssb1-418*, greatly alleviated the repair and mitotic defects of *cut14-Y1*. Ssb1 protein formed nucleolar foci in *cut14-Y1* cells, but the number of foci was diminished in *cut14-Y1 ssb1-418* double mutants. Consistent with the above results, Ssb1 protein bound to single-strand DNA was removed by condensin or the SMC dimer through DNA reannealing *in vitro*. Similarly, RNA hybridized to DNA may be removed by the SMC dimer. Thus, condensin may wind up DNA strands to unload chromosomal components after DNA repair and prior to mitosis. We show that 16 suppressor mutations of *cut14-Y1* were all mapped within the hinge domain, which surrounded the original L543 mutation site.

## 2. Introduction

Condensin is a hetero-pentameric protein complex in eukaryotes that consists of two structural maintenance of chromosomes (SMC) subunits and three regulatory non-SMC subunits [1–6]. The terminal globular domains of the SMC subunits contain the Walker A and B ATPase motifs [7,8], and are connected to a coiled-coil domain that is interrupted by a central hinge, while one of the non-SMC subunits is phosphorylated by Aurora B kinase [9–12] during mitosis [13,14]. The diverse roles of condensin in chromosome dynamics, including mitotic chromosome condensation and segregation, DNA metabolism and development, are well documented [15–19], but the molecular mechanism of how it functions is not well understood. Condensin and the SMC2/4 dimer possess DNA reannealing activity, an activity not found in cohesin [20,21], suggesting that DNA reannealing activity might be required for condensin function. However, the physiological significance of the reannealing activity remains an enigma. In this study, we show that condensin antagonizes replication protein A (RPA) [22–25] activity by removing it from DNA *in vitro* and *in vivo*, which suggests that the DNA reannealing activity of condensin may facilitate the removal of proteins from chromosomes after DNA repair or prior to chromosome segregation.

## 3. Results

### 3.1. Isolation of a structural maintenance of chromosomes mutant hypersensitive to DNA damage

**Figure 1.**
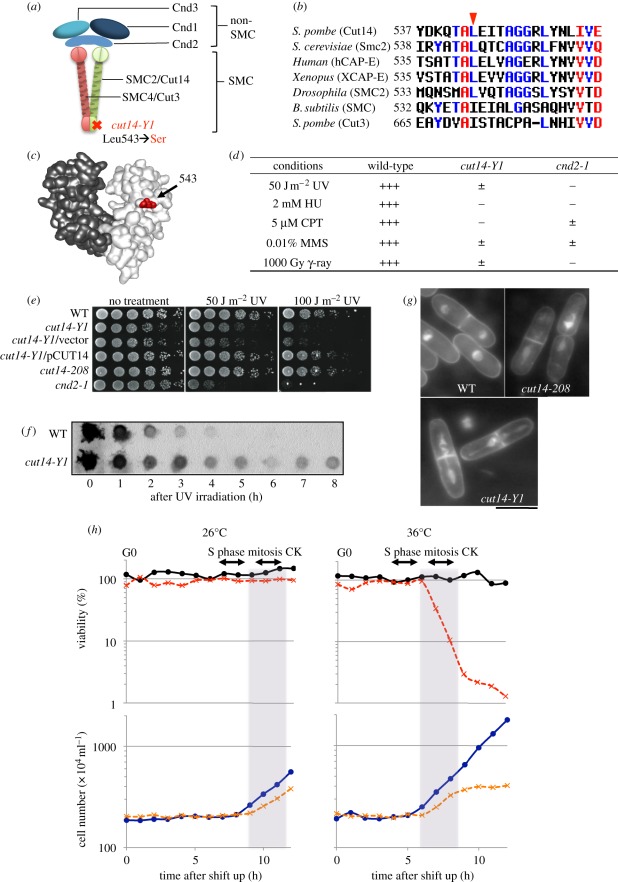
Mutation site and phenotypes of condensin SMC mutant *cut14-Y1*. The identification of *cut14-Y1*: four mutant strains among 1300 *ts* strains examined exhibited condensation defects. Gene cloning, genetic analysis and gene sequencing established that the mutations resided in three distinct genes involved in chromosome condensation. Strain 393 was a DNA topoisomerase II *top2* [26] mutant, strain 640 was a *cut15* [27] (homologue of importin alpha) mutant and the remaining Y1 and 541 strains were *cut14* [28,29] (SMC2 homologue) mutants. Since the *cut14-Y1* strain was hypersensitive to DNA damage at 26°C (the permissive temperature), we examined whether the damage-sensitive phenotype was linked with the *ts* phenotype. Tetrad dissection demonstrated that the HU (hydroxyurea) and UV (ultraviolet) ray-sensitive phenotype co-segregated with the *ts* phenotype (electronic supplementary material, figure S1). (*a*) Heteropentameric condensin complex. The *cut14-Y1* allele consists of a L543S substitution in the hinge. (*b*) Amino acid sequences of the SMC hinge that surround the mutation site (red arrowhead). (*c*) The mutation site (red) is shown within the three-dimensional structure of the mouse hinge domain [30]. (*d*) Summary of the DNA damage phenotypes of *cut14-Y1* together with the previously reported response of *cnd2-1* [31]. +++, normal growth; ±, very slow growth; −, no growth. (*e*) Wild-type (WT), *cut14-Y1* and other strains were spot tested after UV irradiation at 26°C. (*f*) After UV irradiation (100 J m^−2^) at 26°C, extracts of the WT and *cut14-Y1* cells harvested at intervals were immunoblotted using anti-thymine dimer antibodies. (*g*) The mitotic segregation defect of *cut14-Y1* and *cut14-208*. DAPI was used to stain DNA. Scale bar, 10 µm. (*h*) WT and *cut14-Y1* cells were first arrested at the pre-replicative G0 phase in nitrogen-deficient medium (EMM2-N) [32] at 26°C for 24 h, and then shifted to a nitrogen-replenished medium (EMM2) at 26°C (left) or at 36°C (right) for 12 h to measure cell viability (plated at 26°C) and cell number. The timing of S phase, mitosis and cytokinesis (CK) were determined by FACScan and DAPI-staining, respectively. Aliquots of the cultures were taken at 1 h intervals after replenishment, and 300 cells of each genotype were plated on three YPD plates for each time point. The plates were incubated at 26°C for 5 days, and the colony numbers were counted. Circles with solid line, WT; crosses with dashed line, *cut14-Y1*.

About 1300 temperature-sensitive (*ts*) haploid fission yeast *Schizosaccharomyces pombe* strains were constructed, and screened for cytological defects at 36°C (the restrictive temperature) that resembled previously isolated condensation-defective mutants. Identification of a novel condensation-defective *cut14-Y1* mutant is described in the legend of figure 1 and electronic supplementary material, figure S1. This mutant is also highly sensitive to DNA damage at 26°C, the permissive temperature. The hydroxyurea (HU) and ultraviolet (UV) ray-sensitive phenotype co-segregated with the *ts* phenotype. We decided to focus on this mutant, which is the first condensin SMC mutant sensitive to DNA damage to our knowledge. Previously, *cnd2-1*, the Barren-like non-SMC subunit mutant of *S. pombe*, was shown to be sensitive to DNA damage [31].

### 3.2. *cut14-Y1* is a hinge mutant

The *cut14-Y1* mutant contained a single amino acid substitution in the hinge region (L543 to S543; figure 1*a*). Sequence comparisons showed that this amino acid residue is conserved in SMC2 of other organisms, and similar in Cut3/SMC4 (from L to I; figure 1*b*). While the L543 residue is not conserved in the hinge of *Escherichia coli* MukB, this residue is similar in *Bacillus subtilis* SMC (from L to I). Therefore, MukB is probably distinct from that of condensin [33], while *B. subtilis* condensin contains the hinge region, which may be similar to that of eukaryote condensins [34,35]. The L543 residue (red colour in figure 1*c*) is located in the middle of the hinge region and not in the dimerization domain.

### 3.3. DNA damage repair is defective in *cut14-Y1* at 26°C

The response of *cut14-Y1* to various DNA damage agents at 26°C is summarized in figure 1*d* (data are shown in figure 1*e* and electronic supplementary material, figure S2). *cut14-Y1* cells were sensitive to 50 J m^−2^ UV irradiation, 2 mM HU, 5 µM camptothecin (CPT), 0.01 per cent methylmethanesulphonate (MMS) and 1000 Gy*γ*-irradiation, and were more sensitive to CPT than *cnd2-1*. Defects in excision repair were assessed by using an antibody to detect thymine dimers produced by UV for 0–8 h at 26°C. In *cut14-Y1* cells, repair after UV exposure (100 J m^−2^) occurred initially, but was considerably delayed later (figure 1*f*). The damage phenotype of *cut14-Y1* differed greatly from a previously isolated mutant, *cut14-208*, which contains a mutation in the coiled-coil region and is not sensitive to damage [20,31].

### 3.4. Lethal mitosis of *cut14-Y1* occurs at 36°C without delay

A culture of asynchronous *cut14-Y1* mutant cells grown at 26°C was shifted to 36°C. One to two hours after the temperature shift, mutant cells stained with 4′,6-diamidino-2-phenylindole (DAPI) revealed mitotic defects (approx. 100% after 3 h), including abnormal chromosome condensation, segregation and cytokinesis (CK; figure 1*g*). The mitotic defects of *cut14-208* were indistinguishable from those of *cut14-Y1*.

Wild-type (WT) and *cut14-Y1* cells synchronously released from the quiescent G0 phase (figure 1*h*; see the legend for experimental details). The viability of *cut14-Y1* cells at 36°C was identical to that of WT (approx. 100%) during S phase (4 h) and G2 phase, but decreased when cells entered mitosis and CK after 6–8 h at 36°C (dotted red line), suggesting that the lethality occurred at the restrictive temperature after mitotic entry. Potentially lethal defects that occurred before mitosis at 36°C could be rescued if cells were shifted back to 26°C. At 26°C, the viability of *cut14-Y1* was high (100%) and the doubling time was slightly longer than that of the WT. At 36°C, the increase in *cut14-Y1* cell number occurred only once, as cells were dead after the first mitosis (aberrant ‘cut’ cells were counted as two cells). As at 26°C, the timing of CK at 36°C, the restrictive temperature, was only slightly delayed compared with that of WT, suggesting that neither the DNA damage nor mitotic checkpoint were activated in *cut14-Y1*.

### 3.5. *cut14-Y1* genetically interacts with many DNA metabolism mutations

**Figure 2.**
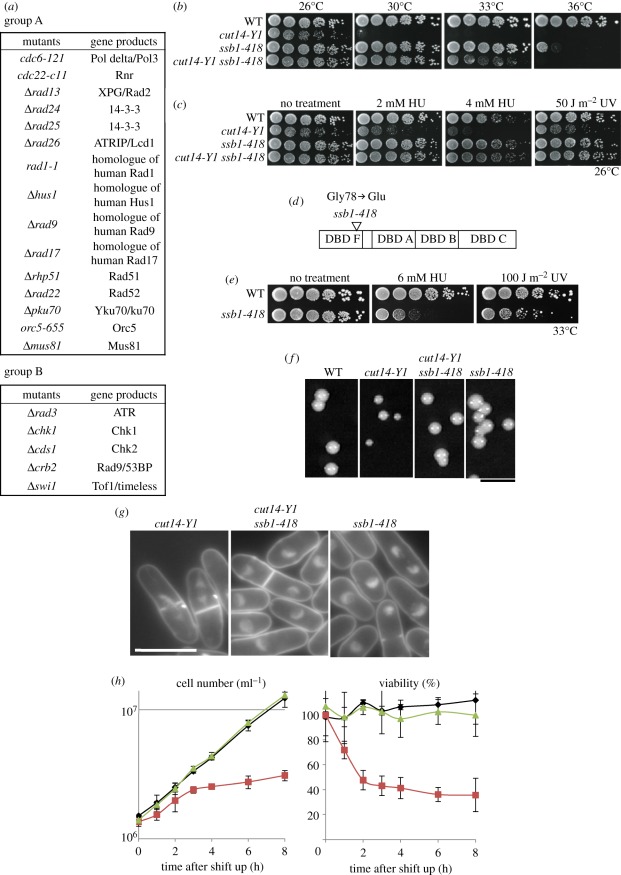
*cut14-Y1* defects are additive with many DNA metabolic and checkpoint mutants, and are rescued by *ssb1-418*. (*a*) Strains crossed with *cut14-Y1* to obtain the double mutants are shown. Crosses with group A (15 strains) did not yield viable double mutants, while those with group B (5 strains) produced double mutants with additive defects. Crosses with group C (14 strains) produced double mutants that did not display any additive defect (see also electronic supplementary material, figure S3). Group A included mutants that are involved with the 9-1-1 complex (*rad9, rad1* and *hus1*), double strand break repair (*rhp51, rad22, mus81* and *ku70*), replication (*cdc6, cdc22* and *orc5*), 14-3-3 (*rad24* and *rad25*), ssDNA nuclease (*rad13*) and DNA damage checkpoint (*rad17* and *rad26*), many of which interact with single-stranded (ss) DNA or ssDNA-associated RPA [36,37]. Group B included five DNA checkpoint mutants (*rad3*, *chk1, cds1, crb2* [38] and *swi1* [39]), suggesting that the hinge of Cut14 might have a checkpoint function. Most of the group C mutants were related to replication, cell cycle and mitosis, although two were involved in DNA repair (*uvde* and *rqh1* [40]). (*b*) *ssb1-418* showed a striking synthetic rescue of the *cut14-Y1 ts* phenotype at 30°C and 33°C. (*c*) *ssb1-418* also rescued the 2−4 mM HU and 50 J m^−2^ UV sensitivity of *cut14-Y1* at 26°C. (*d*) The *ssb1-418* strain contains a G78E amino acid substitution in the DBD F. (*e*) Single *ssb1-418* mutants were sensitive to HU and UV at the semi-permissive temperature (33°C). (*f*) The colony formation of single and double mutants was examined at 26°C. Scale bar, 5 mm. The colony size of *cut14-Y1* single mutants was smaller than that of the *cut14-Y1 ssb1-418* double mutant at 26°C. The doubling time of single *cut14-Y1* was 4.5 h at 26°C, while that of the double mutant and the WT was 3.5 h at 26°C. (*g,h*) In YPD liquid medium at 33°C, the *cut14-Y1* single mutant lost viability and displayed frequent mitotic defects, while the double mutant and *ssb1-418* grew normally. The asynchronous cultures of *cut14-Y1*, *ssb1-418* and the double mutant in the YPD liquid medium were shifted from 26°C to 30°C. The cell number (per millilitre) was counted by the cell counter. The viability was measured at each time point by plating 300 cells spread on YPD plates, incubated at 26°C for 5 days, and resulting colonies were counted. Scale bar, 10 µm. (*h*) Black diamonds, *ssb1-418*; red squares, *cut14-Y1*; green triangles, *cut14-Y1 ssb1-418*.

To understand the function of the condensin hinge domain, synthetic genetic interactions were examined by pair-wise crosses between *cut14-Y1* and 34 mutants defective in cell cycle, mitosis, DNA repair, replication, recombination or the DNA damage checkpoint. Results were classified into three groups (A, B and C), as shown in figure 2*a* and electronic supplementary material, figure S3. Group A mutants, when combined with *cut14-Y1*, failed to produce double mutants by tetrad analysis, suggesting that group A mutants were synthetic lethal with *cut14-Y1*. When *cut14-Y1* was crossed with group B mutants, viable double mutants were produced, but the defects (*ts*, HU and UV) were additive (electronic supplementary material, figure S4). Group C mutants formed viable double mutants with *cut14-Y1*, and the *ts* and HU sensitivity were indistinguishable from that of the *cut14-Y1* single mutant. Hence, we observed genetic interactions with group A and B mutants, with the greatest synthetic interactions observed with group A mutants, whereas group C mutants did not interact with *cut14-Y1*. Group A included mutants, many of which interact with single-stranded (ss) DNA or ssDNA-associated RPA [36,37]. Group B consisted of DNA checkpoint mutants [38], suggesting that the hinge of Cut14 might affect a damage checkpoint function. Most of the group C mutants were related to cell cycle, although two, *Δuvde and Δrqh1*, were involved in DNA repair (electronic supplementary material, figure S3).

### 3.6. Synthetic rescue of *cut14-Y1* by *ssb1-418*

After examining more mutants involved in DNA and RNA metabolism by tetrad dissection, we found that one mutant, *ssb1-418*, showed a striking synthetic rescue of the *cut14-Y1 ts* phenotype at 33°C, and also the HU and UV sensitivities at 26°C (figure 2*b*,*c*). *ssb1-418* is a mutant of Ssb1, the largest subunit of heterotrimeric RPA.

*ssb1-418* was isolated as a *ts* mutant that was suppressed by a plasmid carrying the *ssb1*^+^ gene. The mutation site, determined by genetic crossing and gene sequencing, consisted of an E substitution at G78, and is located in the amino-terminal ssDNA-binding domain called DBD F, which ensures binding to short stretches of ssDNA [41] (figure 2*d*). *ssb1-418* was sensitive to HU (6 mM) and UV (100 J m^−2^) at 33°C, a semi-permissive temperature (figure 2*e*), but not at 26–30°C, suggesting that DNA repair is impaired in *ssb1-418* at higher temperatures.

As shown in figure 2*f*, the colony size of *cut14-Y1* is small, while the colony size of the *cut14-Y1 ssb1-418* double mutant was similar to that of the WT at 26°C. The *cut14-Y1* single mutant frequently exhibited aberrant chromosome segregation, but mitosis in the double mutant appeared mostly normal at 30°C (figure 2*g*). In liquid culture, the double mutant grew exponentially after a temperature shift from 26°C to 30°C (green line, figure 2*h*), while the *cut14-Y1* single mutant arrested after one round of division and quickly lost viability at 30°C (red lines in figure 2*h*). Thus, rescue occurred at the level of mitotic chromosome segregation and cell viability.

### 3.7. Ssb1 nuclear foci are observed in *cut14-Y1* cells, but not in the double mutant

Synthetic rescue suggested that Ssb1 and the Cut14 hinge domain antagonize each other, and that their functional balance might be important. While Ssb1 is known to stabilize DNA strand separation, the Cut14 hinge promotes DNA annealing. Therefore, Ssb1 and condensin may coordinate the dynamics of ssDNA stabilization and destabilization in chromatin. To test whether Ssb1 might anomalously remain in mitotic chromatin in the *cut14-Y1* mutant, localization of Ssb1 was assessed by immunofluorescence microscopy using polyclonal antibodies against *S. pombe* Ssb1 [42].

**Figure 3.**
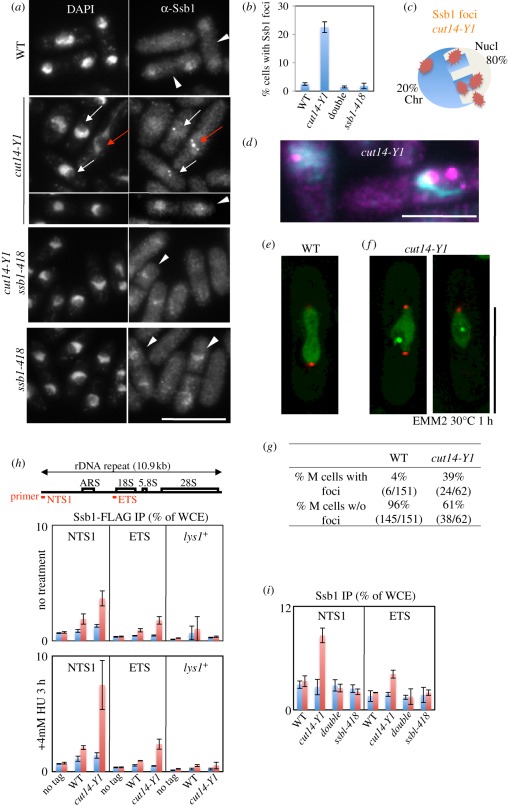
Intense Ssb1-YFP foci formed in the nucleolus of *cut14-Y1*, but not in the double mutant. (*a*) Intense nuclear foci were detected by anti-Ssb1 antibody (DNA stained by DAPI). Immunofluorescence micrographs are shown for WT, single *cut14-Y1*, *ssb1-418* and the double mutant cells that were shifted from 26°C to 30°C for 1 h. The Ssb1 foci were observed in both mitotic (red arrows) and interphase (white arrows) *cut14-Y1* cells. Such foci were scarce in WT, *ssb1-418* and *cut14-Y1 ssb1-418* (double mutant) cells. The nuclear Ssb1 signals were clearly observed in S phase cells (white arrowheads). The S phase occurs in binucleated septated cells, allowing S phase cells to be easily distinguished. In those S phase cells, nuclear accumulation of Ssb1 appeared as punctate signals, distinct from the foci observed in *cut14-Y*1. Scale bar, 10 µm. (*b*) Quantitative data (percentage cells with Ssb1 foci) are shown. Three hundred cells were observed for each strain. (*c*) Anti-Ssb1 antibodies revealed that the intense foci were mostly (approx. 80%) located in the nucleolar region of the *cut14-Y1* single mutant. The *S. pombe* interphase nucleus consists of the hemispherical chromatin region (Chr) and the remaining nucleolar (Nucl) region [43]. (*d*) The enlarged, merged micrograph of *cut14-Y1* cells. Blue, DAPI; purple, Ssb1. Scale bar, 5 µm. (*e,f*) Live cell images of WT (*e*) and *cut14-Y1* mutant (*f*) cells that express both Ssb1-YFP (green) and Sad1-mCherry (SPB, red). Scale bar, 10 µm. See electronic supplementary material, movies S1–S4. (*g*) The frequency (%) of cells showing Ssb1-YFP foci in WT and *cut14-Y1* mitotic mutant cells were determined after the shift to 30°C (the restrictive temperature) from 26°C for 1 h in EMM2. (*h*) Chromatin immunoprecipitation (ChIP) experiment using anti-FLAG antibodies. The *ssb1*^+^ gene was chromosomally tagged with FLAG in the WT and *cut14-Y1* strains, and expressed under the native promoter at 26°C in the absence (upper panel) or presence (lower panel) of 4 mM HU for 3 h. Two rDNA probes and one negative control (*lys1*^*+*^) probe were used. Blue and red columns indicate ChIP without and with antibodies against FLAG. An untagged (no tag) strain was used as the negative control. (*i*) ChIP experiment using anti-Ssb1 antibodies for the four strains cultured at 26°C. (*h,i*) Blue bars, −antibodies; red bars, +antibodies.

Anti-Ssb1 antibody revealed that the nuclear Ssb1 signals were clearly observed in S phase cells. In those S phase cells, Ssb1 appeared as punctate signals in 100 per cent of cells (arrowheads in figure 3*a*). In *cut14-Y1* mutant cells or the other mutants, *ssb1-418* and *ssb1-418 cut14-Y1*, at the permissive temperature, the Ssb1 dots in S phase were similarly observed in 100 per cent of cells (arrowheads) and disappeared in G2 phase. These Ssb1 signals in S phase are distinct from the foci observed in *cut14-Y1* (see below).

Intense nuclear foci, as detected by anti-Ssb1 antibody (DNA stained by DAPI), were frequently observed in both mitotic (red arrow, figure 3*a*) and interphase (short white arrows) *cut14-Y1* cells. Such foci were scarce in WT, *ssb1-418* and *cut14-Y1 ssb1-418* (double mutant) cells. We measured the frequency of cells exhibiting these nuclear foci and, as shown in figure 3*b*, 22 per cent of the *cut14-Y1* cells contained such foci, whereas the foci were infrequent (less than 2%) in the other three strains. This result suggested that foci formation in *cut14-Y1* required the presence of WT Ssb1.

We then examined the localization of these intense foci of Ssb1 in *cut14-Y1* mutant cells. The majority (80%) of foci were located in the rDNA nucleolar region, while the remaining foci (20%) resided at the periphery of the nucleus (figure 3*c*; the illustration shows the location of Ssb1 foci in *cut14-Y1*). Figure 3*d* shows multiple nucleolar foci in the right cell and one peri-nuclear focus in the left cell.

### 3.8. Live cell analysis of Ssb1-YFP foci in *cut14-Y1* mitotic cells

To further investigate the results above, we constructed strains carrying chromosomally integrated Ssb1-YFP that was expressed from its native promoter, and observed Ssb1-YFP signals in WT or *cut14-Y1* living cells. Ssb1-YFP was also located in the mitotic nucleoli in WT and in *cut14-Y1* (figure 3*e*,*f*). In the electronic supplementary material movies, the dynamics of Ssb1-YFP signals in mitosis followed by S phase from WT and *cut14-Y1* cells are shown. The YFP signals in WT were smoothly located in the nuclear chromatin region during mitosis (figure 3*e*) and became punctate during S phase (electronic supplementary material, movie S1). In *cut14-Y1* cells, intense Ssb1 foci were observed in the nucleolar region of aberrantly elongated mitotic chromosome (figure 3*f* and electronic supplementary material, movies S2–S4).

After analysing a large number of movies, Ssb1-YFP foci were found in 39 per cent (24/62) of mitotic *cut14-Y1* cells, but only in 4 per cent (6/151) of the WT mitotic cells (figure 3*g*). Movies clearly showed that the *cut14-Y1* mutant cells containing foci were not delayed prior to the entry into mitosis, suggesting that the mitotic checkpoint was not activated (see below).

### 3.9. Ssb1 accumulated in the rDNA region of *cut14-Y1*, but not in the double mutant

Chromatin immunoprecipitation (ChIP) of rDNA non-coding regions was performed using chromosomally integrated Ssb1-FLAG expressed from its native promoter. Antibodies against FLAG were used for ChIP of cells cultured in the presence or absence of 4 mM HU. As shown in figure 3*h*, more Ssb1 bound to rDNA (primer NTS1) in *cut14-Y1* cells cultured at 26°C compared with WT. In the presence of HU, the level of Ssb1 bound to rDNA increased in *cut14-Y1*, indicating that more Ssb1 was bound to rDNA in *cut14-Y1*.

To analyse the level of Ssb1-418 mutant protein that bound to rDNA, polyclonal antibodies against Ssb1 were used. As shown in figure 3*i*, the level of Ssb1 mutant protein bound to rDNA was diminished in the *cut14-Y1 ssb1-418* double mutant when cultured at 26°C. This defect of the Ssb1-418 protein may explain why the *ssb1-418* mutant suppresses the phenotypes of *cut14-Y1*.

### 3.10. Ssb1-YFP foci are also observed in *cnd2-1* cells but not in mitotic cells

**Figure 4.**
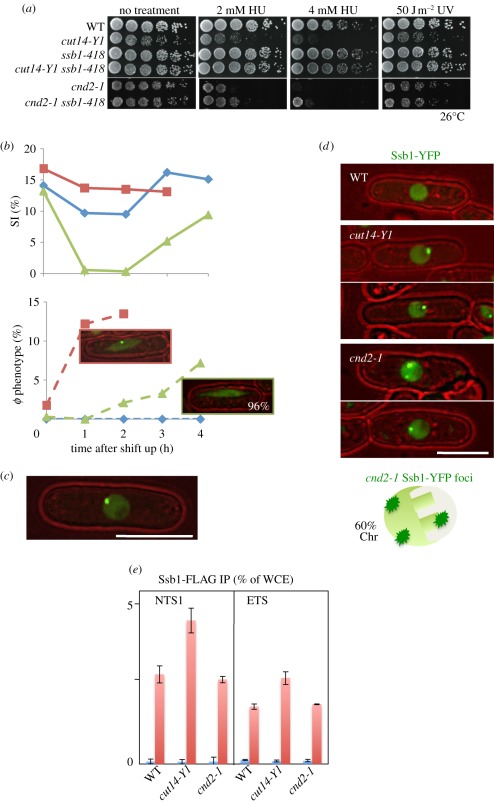
The checkpoint response and nuclear localization of Ssb1-YFP differ in *cnd2-1* and *cut14-Y1*. (*a*) Whereas the DNA damage sensitivities of *cut14-Y1* were greatly rescued by *ssb1-418* mutation, those of *cnd2-1* were not. (*b*) WT, *cut14-Y1* and *cnd2-1* were cultured at 36°C for 0–4 h, and the percentage septation index (SI) and the number of cells displaying aberrant chromosome (*φ* phenotype) were measured. The frequency of aberrant mitotic chromosomes sharply increased in *cut14-Y1* (blue diamonds with solid line, WT; red squares with solid line, *cut14-Y1*; green triangles with solid line, *cnd2-1*; top), while the appearance of such mitotic cells was delayed in *cnd2-1* (blue diamonds with dashed line, WT; red squares with dashed line, *cut14-Y1*; green triangles with dashed line, *cnd2-1*; bottom). Notably, the aberrant mitotic chromosomes in *cnd2-1* did not contain Ssb1 foci (96%). (*c*) The intense foci of Ssb1-YFP were observed in *cnd2*-*1* cells. Note that the YFP dot (the focus) is located in the nuclear periphery chromatin region. (*d*) Distinct nuclear localization of the Ssb1-YFP signals in *cnd2-1* and *cut14-Y1*. The WT cell nucleus did not show the foci of Ssb1-YFP. Two *cut14-Y1* cells display the intense Ssb1-YFP foci, which are located in the nucleolar region. Two *cnd2-1* cells also show the intense Ssb1-YFP foci, which are located in the non-nucleolar nuclear chromatin region. Sixty per cent of cells examined showed the nuclear chromatin localization of Ssb1-YFP foci in *cnd2-1* mutant cells. (*e*) ChIP experiment of Ssb1-FLAG for the rDNA probe NTS1 using WT, *cut14-Y1* and *cnd2-1* strains. The procedures are the same employed in figure 3*h*. Blue bars, −antibodies; red bars, +antibodies.

Non-SMC mutant *cnd2-1* was also DNA damage sensitive [31]. However, the phenotypes of *cnd2-1* were not rescued by *ssb1-418* (figure 4*a*). The reason for this failure of suppression might be due to the loss of the Ssb1-YFP foci in mitotic *cnd2-1* cells (described below). When the temperature of the *cnd2-1* culture was shifted from 26°C to 36°C, the septation index (SI) declined rapidly owing to activation of the Cds1-dependent checkpoint [31], whereas no decrease of the SI occurred in *cut14-Y1* (figure 4*b*). The results (explained in the legend) suggest that at 36°C, the DNA checkpoint was activated in *cnd2-1*, but not in *cut14-Y1*, and the *cnd2-1* cells that entered mitosis after the delay did not contain Ssb1-YFP foci. However, the Ssb1-YFP foci were found in about 20 per cent of interphase cells of *cnd2-1* (figure 4*c*).

The Ssb1-YFP foci were located in the non-nucleolar chromatin region of *cnd2-1* mutant cells. Examples of these cells are shown in figure 4*d*. Consistently, ChIP of Ssb1-FLAG shows that Ssb1-FLAG is not enriched in the rDNA in *cnd2-1* cells (figure 4*e*), in sharp contrast to the result in *cut14-Y1*. Taken together, Ssb1 probably binds to non-nucleolar chromosomal regions in *cnd2-1*, leading to the Cds1-dependent DNA damage checkpoint delay.

### 3.11. Purified condensin and structural maintenance of chromosomes dimer preferentially interact with ssDNA

**Figure 5.**
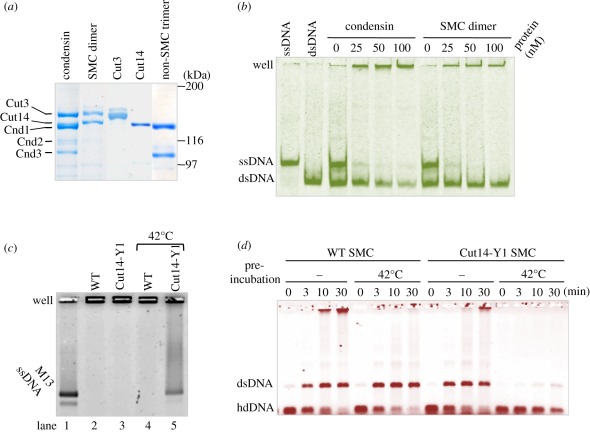
Interaction of isolated condensin and SMC dimer with different DNAs. (*a*) SDS-PAGE patterns of holocondensin (Cut3-Cut14-Cnd1-Cnd2-Cnd3), the SMC dimer (Cut3-Cut14) and the non-SMC trimer (Cnd1-Cnd2-Cnd3), together with single Cut3 and Cut14 as controls, stained with Coomasie brilliant blue. The procedures of isolation were previously described, and the degree of purity for these preparations was similar to those previously reported [20,21]. The Cut14 and Cnd1 overlap, and the Cnd2 band is diffuse and less intense than the other non-SMC subunits, probably owing to phosphorylation and/or degradation [9]. Limited proteolysis of Cut3 has been reported [20]. (*b*) Condensin and SMC dimer were incubated with a mixture of ssDNA and dsDNA, then analysed on a 10% non-denaturing acrylamide gel in the absence of SDS. DNA used was tagged with fluorescent FITC. (*c*) WT and mutant SMC dimer were incubated with M13 ssDNA with or without the pre-heat treatment at 42°C for 10 min, then analysed on a 0.7% native agarose gel in the absence of SDS. The mutant dimer was obtained by simultaneous overexpression of Cut3 and Cut14-Y1, and purified by affinity chromatography, stained with SYBR Gold. (*d*) WT and mutant SMC dimers were incubated with hdDNA with or without pre-heat treatment of the SMC dimers (see text), stained with ethidium bromide.

To examine DNA–protein interactions, we purified condensin protein complexes from *S. pombe* cells where all the condensin subunits were simultaneously overexpressed [20,21]. Protein preparations were run on a sodium dodecyl sulphate polyacrylamide gel electrophoresis (SDS-PAGE) gel and stained with Coomasie brilliant blue (figure 5*a*).

Holocondensin and the SMC dimer were incubated with short ssDNA (86 nucleotide long) and dsDNA (86 bp) for 10 min at 30°C. Although both bound to ssDNA and dsDNA, they bound preferentially to ssDNA, as seen in the acrylamide native gel (figure 5*b*). The band intensity of ssDNA decreased greatly when increasing amounts of condensin or SMC dimer were added. The SMC dimer appeared to have a stronger affinity for ssDNA than holocondensin. The associated protein–DNA complex did not produce a band shift, but instead remained in the well owing to the aggregate formation of DNA–protein [21].

### 3.12. Weak DNA binding and reannealing by the mutant structural maintenance of chromosomes dimer protein

We then examined whether the mutant Cut14-Y1 protein was similarly capable of binding DNA, by using a Cut3-Cut14-Y1 heterodimer. M13 phage ssDNA (7.2 kb long) was purchased (NEB) and used for the DNA–protein binding experiments. As seen in figure 5*c*, M13 ssDNA (producing two bands) moved to the top of the native agarose gel, which had no sodium dodecyl sulphate (SDS) added, when mixed with the WT and mutant SMC dimer (lanes 2 and 3). If the protein preparations were pre-treated with heat (42°C for 10 min [20]), M13 ssDNA associated with the WT SMC complex still remained at the top of gel (lane 4), whereas the mutant Cut3-Cut14-Y1 dimer produced smeared bands from the position of the M13 ssDNA band to the gel top (lane 5), suggesting that the complex formation of the mutant dimer on ssDNA was greatly diminished after heat treatment.

We then compared the DNA reannealing activity of WT and mutant SMC dimer with or without heat pre-treatment. As shown in figure 5*d*, WT and mutant (Cut14-Y1) SMC dimers with (42°C) or without (−) pre-heat treatment were incubated with heat-denatured (hd) DNA (single cut linear 3.0 kb blue script), then the reactions of hdDNA with SMC at 30°C for 0–30 min were stopped by the addition of 0.2 per cent SDS. At 0 min, only hdDNA was observed. The WT SMC dimer promoted annealing to produce dsDNA. Upon heat pre-treatment of the WT SMC dimer, a dsDNA band was also formed. Without heat treatment, the mutant SMC dimer could form dsDNA, but completely failed to reanneal hdDNA upon heat treatment.

### 3.13. DNA reannealing promotes the release of replication protein A bound to heat-denatured ssDNA

**Figure 6.**
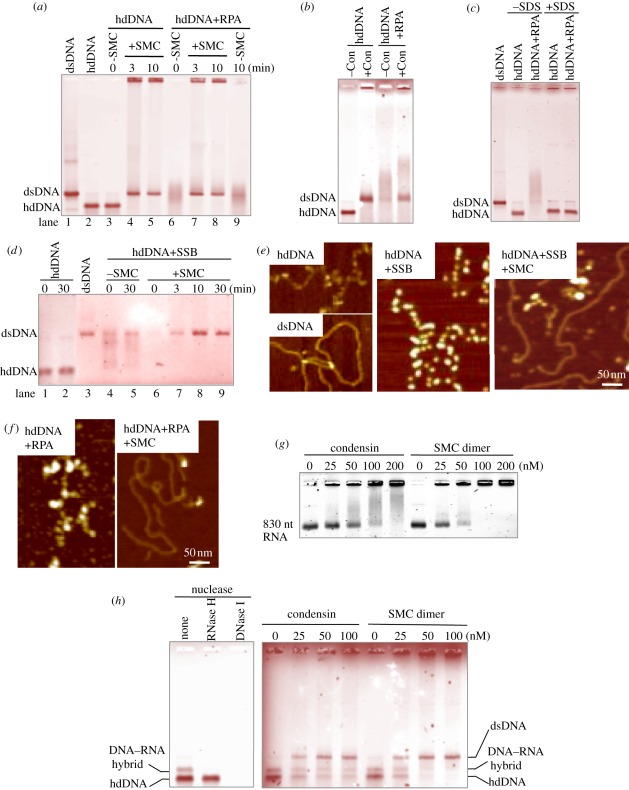
Condensin SMC-mediated elimination of RPA from hdDNA. (*a*) SMC dimer promotes reannealing of RPA-coated hdDNA. Lanes 1,2: control ds and hdDNA; 3–5: naked hdDNA (heat denatured and then rapidly cooled) was incubated with or without SMC for 0, 3 or 10 min; 6–9: hdDNA pre-coated with RPA was further incubated with (lanes 6–8) or without (lane 9) the SMC dimers. After incubation, samples were analysed on a 0.7% native agarose gel (without SDS). (*b*) Holocondensin also produced dsDNA from RPA-coated hdDNA. Native agarose gel was used. (*c*) hdDNA incubated with RPA complex was analysed in the absence or presence of SDS. See text. (*d*) Lanes 1,2: hdDNA incubated alone for 0 or 30 min; 3: dsDNA; 4–9: hdDNA pre-coated with SSB for 5 min at 30°C, and further incubated for 30 min without (lanes 4,5) or with SMC for 0–30 min (lanes 6–9). The reaction mixtures were analysed by native agarose gel electrophoresis. (*e*) AFM images hdDNA (top left), dsDNA (bottom left), hdDNA coated with SSB (middle). SMC was added and incubated with SSB-coated hdDNA for 30 min (right). (*f*) AFM images of hdDNA coated with *S. pombe* RPA (left); SMC dimer was added and incubated with RPA-coated hdDNA for 30 min (right). (*g*) Condensin and SMC dimer binding to RNA that was made in electronic supplementary material, figure S5. The samples were analysed using a 4% native agarose (NuSieve) gel in the absence of SDS. (*h*) (left) The mixture of hdDNA and DNA–RNA hybrid was digested with DNase I or RNase H. The hybrid band was selectively digested with RNase H. (right) Condensin and SMC dimers (0–100 nM) were incubated with the mixture, and SDS was used to stop the reactions. The samples were analysed using a 0.7% agarose gel. Staining with (*a–d,h*) ethidium bromide and (*g*) SYBR Gold.

To determine whether the SMC dimer promoted reannealing *in vitro* when complementary ssDNAs were previously coated with purified heterotrimeric RPA [42], hdDNAs were first mixed with *S. pombe* heterotrimeric RPA (producing diffuse bands in the native gel) and then briefly incubated with the SMC heterodimer, yielding a duplex dsDNA band that formed within 3 min (figure 6*a*). Holocondensin also reannealed RPA-coated hdDNA, but less efficiently than the SMC dimer (figure 6*b*).

In order to firmly confirm that the diffuse bands produced by hdDNA and RPA were indeed owing to complex formation between RPA and ssDNA, and not with dsDNA, the complex formed by hdDNA (40 ng) and RPA on ice for 10 min was treated with 1 per cent SDS (+ SDS; figure 6*c*). When the mixture was examined in the absence of SDS (−SDS), smeared bands were observed, but not in the presence of SDS. No dsDNA formation thus occurred in this experiment.

To visualize the removal of RPA from hdDNA, atomic force microscopy (AFM) was used [4,21] (figure 6*d–f*). Highly purified bacterial single-strand DNA binding protein (SSB; purchased from Promega)-coated hdDNA was incubated with the *S. pombe* SMC dimer. Within 10 min, a sharp dsDNA band was formed (figure 6*d*). AFM images of hdDNA before and after the addition of SSB, followed by the addition of the SMC dimer, are shown in figure 6*e*. Beaded ssDNA coated with SSB was observed for hdDNA mixed with SSB (middle), while dsDNA was plentifully produced 30 min after the incubation with SMC dimer (right; control hdDNA and dsDNA images, left top and bottom, respectively).

Purified *S. pombe* RPA was also examined by AFM (figure 6*f*, left). Coated ssDNA similar to that produced by bacterial SSB was observed. Thirty minutes after the incubation with SMC dimer, dsDNA was plentifully observed (figure 6*f*, right). Taken together, our data show that condensin SMC promotes DNA reannealing and releases bacterial SSB or *S. pombe* RPA bound to complementary ssDNA.

### 3.14. Interactions of condensin structural maintenance of chromosomes to RNA and RNA–DNA hybrid

To substantiate these findings in a broader physiological context, condensin binding to RNA was examined. As shown in figure 6*g*, both condensin and the SMC dimer were bound to 830 nt RNA (electronic supplementary material, figure S5) and formed a complex that did not enter the gel, though the SMC dimer was bound to RNA more efficiently. To our knowledge, this is the first demonstration of the interaction of RNA with condensin SMC.

An RNA–DNA hybrid was then constructed by mixing transcribed RNA with hdDNA (described in electronic supplementary material, figure S5) and then adding the SMC dimer or condensin. RNase H was used to verify the hybrid. The SMC dimer reduced the amount of the RNA–DNA hybrid and increased the level of dsDNA, suggesting that condensin SMC might interact with RNA–DNA hybrid and remove RNA, followed by the formation of dsDNA (figure 6*h*; SDS was added before electrophoresis). Another interpretation might be possible, such that condensin SMC might interact with the RNA–DNA hybrid to form the higher-order aggregate.

### 3.15. Ssb1-418 mutant protein exhibits diminished ssDNA binding

**Figure 7.**
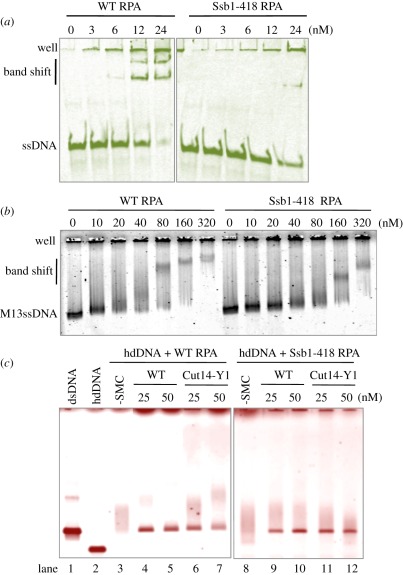
Interaction of mutant RPA with DNA and the mutant SMC dimer. (*a,b*) Interaction of WT and mutant RPA complexes with (*a*) short and (*b*) long ssDNA. The heterotrimeric RPA that contained the Ssb1-418 mutant protein was purified and mixed with (*a*) short 86 nt ssDNA and (*b*) long M13 ssDNA, followed by (*a*) native acrylamide and (*b*) native agarose gel electrophoresis (in the absence of SDS). Binding of the mutant RPA to short 86 nt ssDNA was greatly diminished, whereas the binding to M13 ssDNA was only slightly diminished. (*c*) WT and mutant RPA (80 nM) were bound to heat-denatured hdDNA for 5 min on ice, followed by the addition of WT and mutant SMC dimer-containing Cut14-Y1 (0, 25, 50 nM) for the reannealing reaction at 30°C for 30 min. Resulting reaction mixtures were analysed using 0.7% native agarose gels and stained with ethidium bromide. Diffuse bands represented hdDNA coated with RPA, which formed with the WT and mutant RPA. The ability of mutant SMC dimer (Cut14-Y1) for reannealing was diminished for hdDNA precoated with the WT RPA, whereas the reannealing went equally well when the mutant RPA previously coated hdDNA. Staining with (*a*) FITC, (*b*) SYBR Gold and (*c*) ethidium bromide.

To further examine why *ssb1-418* rescued the phenotypes of *cut14-Y1*, we characterized the properties of the mutant Ssb1 protein. ssDNA binding was tested using short and long ssDNAs. The heterotrimeric RPA containing the Ssb1-418 protein was purified and mixed with ssDNAs. Binding of the mutant RPA to short 86 nt ssDNA was greatly diminished (figure 7*a*), whereas the binding to long 7.2 kb M13 ssDNA was only slightly diminished (figure 7*b*) in comparison with WT RPA.

We then examined whether *cut14-Y1* mutant SMC-mediated RPA removal from ssDNA differed when the WT or mutant RPA was employed. As shown in figure 7*c* (lane 3–7), the WT SMC dimer was more efficient than the mutant SMC dimer for the removal of WT RPA from ssDNA by reannealing. In contrast, when the mutant RPA was employed (lane 8–12), the removal of the mutant RPA from ssDNA was equally efficient by reannealing promoted by the WT and mutant SMC dimer. These results may explain the genetical rescue of the double mutant *cut14-Y1 ssb1-418*.

### 3.16. Intragenic suppressor mutations of *cut14-Y1*

**Figure 8.**
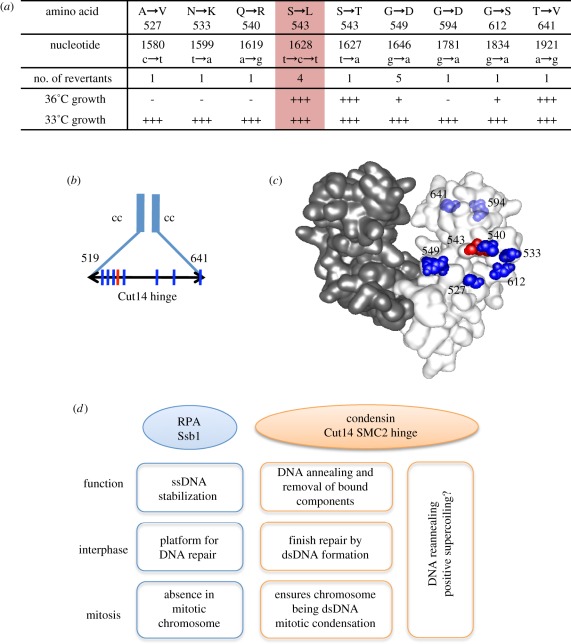
The hinge of SMC2/Cut14 is a functional entity. (*a*) Mapping of pseudo-revertants of *cut14-Y1* that formed colonies at 33°C. The four mutants are true revertants (S543L, red column) that formed the normal colonies at 36°C. See text. (*b*) The mutation sites are shown by the vertical lines. The hinge region is between the residues 519 and 641. cc, coiled-coil. (*c*) Location of the mutation sites in the three-dimensional structure of the mouse condensin hinge [30]. The amino acid residue number is adapted for the *S. pombe* Cut14. The original *cut14-Y1* mutation site is indicated by the red colour, while the second suppressing mutation sites are shown by blue. The residues 594 and 641 situating behind are faded. (*d*) A diagram depicting the relationship between the condensin SMC Cut14 hinge and Ssb1. Condensin preferentially binds to ssDNA [30,34] and promotes annealing to complementary ssDNA *in vitro*, and appears to oppose the action of RPA. RPA acts as a platform for various proteins involved in DNA metabolism, such as damage repair and replication through ssDNA stabilization [44–46]. The role of condensin in damage repair remains unclear, but we propose that it may be required for completing/exiting repair processes by removing RPA and forming dsDNA through reannealing.

We attempted to isolate extragenic suppressors of *cut14-Y1*, and obtained 16 Ts^+^ (at 33°C) suppressors of *cut14-Y1*. Surprisingly, the mutations all mapped to within the *cut14* locus and were all located within the hinge domain (519–641aa; figure 8*a*,*b*). Four were true revertants (S543L) and formed colonies at 36°C. One suppressor also contained a substitution at the same residue (S543T) but, curiously, behaved like the WT. Eleven contained a second site mutation in addition to the original L543S mutation. Based on the three-dimensional structure [30], the additional mutation sites (blue) surrounded the L543 site, but resided largely out of the dimerization domain (red; figure 8*c*). These suppressor mutations appeared to restore the DNA binding and reannealing activity of the hinge. The hinge may thus be a flexible structure in which deleterious mutations can be compensated by the presence of the additional mutations within the hinge domain.

## 4. Discussion

Understanding the mechanisms of how condensin functions in mitosis and DNA repair is of great importance. We identified a fission yeast *ts* mutant *cut14-Y1* that contains a point mutation in the hinge domain of SMC2, a subunit of condensin. This mutant displayed severely reduced viability during mitosis at elevated temperatures and after exposure to DNA damage agents at the permissive temperature. Repair of UV-induced DNA damage is defective. Other phenotypes include a mitotic segregation defect and an inability to reanneal heat-denatured complementary DNAs (hdDNA). Further genetic analysis showed that a mutant of the large subunit of RPA (*ssb1-418*) rescued almost all the *cut14-Y1* phenotypes observed.

Strikingly, we found that nucleolar RPA foci formation and Ssb1 binding to rDNA in *cut14-Y1* were largely reduced in the *cut14-Y1 ssb1-418* double mutant. *In vitro* assays demonstrated that RPA was removed by SMC-promoted reannealing of hdDNA. We propose that condensin may compete with RPA-induced DNA strand separation by re-annealing separate ssDNA strands after DNA repair and prior to mitosis in order to unload chromosomal components. We speculate that the regulation of the non-SMC trimer by Aurora kinase B [9–11] or its interaction with histone H2A [47] during mitosis may be important for the activation of its reannealing activity. *In vitro*, the non-SMC trimer has been shown to inhibit DNA reannealing [21]. Non-SMC trimer may thus be negatively regulated by Aurora kinase B during mitosis.

We previously showed that the non-SMC condensin *cnd2-1* mutant was sensitive to DNA damage and exhibited cell cycle delays owing to DNA repair [31], but it remained possible that Cnd2 might have a role unrelated to condensin. Our present data provide additional convincing evidence supporting the idea that condensin is required for the repair of DNA damage. We also show that the condensin Cut14/SMC2 hinge may be required for damage checkpoint activation. Consistently, the damage checkpoint activation occurs in *cnd2-1* that contains the WT SMC2 hinge.

Our *in vitro* results showed that the SMC dimer and holocondensin promote the removal of DNA-bound RPA through reannealing. This is consistent with our *in vivo* results demonstrating that the Ssb1 foci observed by immunofluorescence and live cell imaging are produced in the *cut14-Y1* hinge mutant cells, which contain aberrant mitotic chromosomes. The striking rescue of *cut14-Y1* by *ssb1-418* suggested that the weakened Ssb1 activity alleviated the defects generated by the Cut14 hinge mutation. Indeed, results from ChIP and *in vitro* reannealing assays suggested that RPA containing the mutant Ssb1-418 subunit may be removed by mutant condensin. As shown in figure 8*d*, we speculate that Ssb1 and Cut14 coordinate to regulate the dynamics of ssDNA stabilization.

The damage repair defect in *cnd2-1* activates damage checkpoint in a Cds1-dependent manner, and delays mitotic entry [31], whereas the damage in *cut14-Y1* is slowly repaired and does not seem to activate the checkpoint. It is possible that the SMC2 hinge may act in parallel with the damage checkpoint. Ssb1 formed intense nucleolar foci in *cut14-Y1*, which may not be recognized by the DNA damage checkpoint and may also not be removed prior to mitosis owing to the condensin defect. By contrast, *cnd2-1* formed similarly intense Ssb1 foci, but the foci were located in non-nucleolar regions. Additionally, the Ssb1-YFP foci did not remain in the aberrant chromosomes of mitotic cells after the delay. This striking difference is consistent with the failure of the rescue of *cnd2-1* by *ssb1-418*.

The regulation of the positive supercoiling activity [48] of condensin by protein kinases such as Cdk1 [49], Aurora B and polo kinases [50], and its relationship to DNA topoisomerase II [51], have been extensively studied. These studies emphasized the role of condensin in chromatin packing, though the mechanism of how the complex functions has not been elucidated. In this study, we present evidence suggesting that condensin may have a function in clearing DNA [19]. The transition from fuzzy chromatin in the interphase nucleus to compacted mitotic chromosomes may require the removal of interphase protein components such as RPA. The SMC hinge appears to be critical for this transition. Cti1/C1D, which directly interacts with the condensin Cut3/SMC4 hinge and rescues *cnd2-1* [52], is required for RNA degradation [53]. Previous structural and mutational analyses showed that the hinge is required for DNA binding and dimerization [5,30,35]. It has been suggested that the cohesin hinge may open upon DNA binding [54–56]. Based on our results, the hinge appears to be important for binding with DNA and RNA, DNA annealing, damage repair, elimination of protein and RNA from DNA, and chromosome condensation. Thus, uncovering how the condensin hinge functions may be key to understanding the many roles of condensin.

RPA and condensin appear to antagonize each other, but a balance in their activities may be important for coordinating the dynamics of strand separation. Prior to mitosis, RPA may need to be excluded from chromosomes, and condensin may remove RPA or possibly other components such as transcribed RNA to ensure that mitotic chromosomes are properly formed and maintained. The promotion of DNA reannealing by condensin SMC involves the winding up of DNA strands, which is similar to the formation of positive supercoiling (figure 8*d*). Condensin may supercoil DNA strands to unload chromosomal components after DNA repair and prior to mitosis. Our present finding that condensin winds up DNA to clear the DNA-bound components may provide a physiological meaning of SMC-mediated DNA reannealing. Instead, unwinding the double-stranded DNA leads to the formation of negative supercoil, which results in the association of transcription machinery and replication apparatus to unwound chromosomal DNA regions. This hypothesis will require further investigation.

## 5. Material and methods

### 5.1. Isolation of *cut14-Y1* and *ssb1-418*

These two strains were isolated by screening the *S. pombe ts* strain collection made by random mutagenesis using procedures similar to those described previously [57]. *cut14-Y1* was isolated owing to its cytological defect in mitotic condensation, while *ssb1-418* was rescued by plasmids carrying the *ssb1*^+^ gene. Gene cloning and tetrad dissection confirmed the tight genetic linkage of the *cut14-Y1 ts* phenotype to the *cut14* locus, whereas *ssb1-418* is caused by a mutation in the *ssb1* locus. The mutation sites of both *cut14-Y1* and *ssb1-418* (G78E) were determined by nucleotide sequencing of the mutant genes isolated by PCR.

### 5.2. Media, strains and plasmids

Yeast extract, polypeptone, d-glucose (YPD), sporulation agar (SPA) sporulation and minimal Edinburgh minimal medium 2 (EMM2) media were used for culturing *S. pombe* cells. The pre-replicative G0 cells made under nitrogen starvation conditions were replenished by the addition of 0.5 per cent NH_4_Cl [32]. The chromosomally integrated strain of YFP-tagged Ssb1 strain expressed under the native promoter was made as described [28]. The Sad1-mCherry was used as the spindle pole body (SPB) marker [58]. Plasmids used in this study are shown in electronic supplementary material, table S1.

### 5.3. Visualization of mutation sites in the three-dimensional structure

The images of mutation sites in the three-dimensional structure were made using molecular structure visualization software (MolFeat; FiatLux). The mouse SMC2-4 structure [30] was obtained from the RCSB Protein Data Bank (http://www.pdb.org/pdb/home/home.do).

### 5.4. DNA damage sensitivity, ultraviolet irradiation and thymine dimer detection

The procedures for UV irradiation, HU and other drug sensitivity measurements, and the detection of thymine dimers, were previously described [31].

### 5.5. Immunofluorescence microscopy and chip

The procedures for DAPI staining and immunofluorescence microscopy were previously described [58]. The anti-Ssb1 antibody was previously made and characterized [42]. The ChIP method was performed as previously described [58], with slight modifications. Immunoprecipitation was performed using anti-FLAG M2 antibody (Sigma-Aldrich) or anti-Ssb1 antibody. Real-time PCR was performed on the Exicycler (Bioneer). The PCR primers used were previously described [58].

### 5.6. Live cell analysis

Live cell analysis (figure 3*e–g*; electronic supplementary material, movies S1–S4) was performed as previously described [58]. In brief, *S. pombe* cells were cultured at 26°C in EMM2 medium and were shifted to 30°C for the appropriate duration. Cells were transferred to a glass-bottomed dish (IWAKI Glass) coated with concanavalin A (Wako) before being examined under the microscope (DeltaVision; Applied Precision). Time-lapse images were recorded by three-dimensional microscopy using the DeltaVision system. For imaging of the Ssb1-YFP with Sad1-mCherry, three optical sections were collected at 0.5 min intervals at 30°C. The vertical separations between these sections were 0.5 µm. Image projection and deconvolution were performed using an imaging workstation (SoftWoRx; Applied Precision). Video images (electronic supplementary material, movies S1–S4) were taken at 0.5 min intervals. The display speed is 3 frames s^−1^.

### 5.7. Protein purification and DNA annealing assay

*Schizosaccharomyces pombe* single condensin subunits, heteropentameric holocondensin, dimer or trimer subcomplexes [20,21] and the heterotrimeric RPA complex [42] were purified as described. Bacterial SSB was purchased from Promega. DNA annealing assay (in the absence of RPA or SSB) was performed as described [20]: the reactions were stopped by the addition of SDS (final 0.2%). For the protein (RPA or SSB) elimination assay, 40 nM *E. coli* SSB or 40 nM *S. pombe* heterotrimeric RPA was used for the precoating of hdDNA (linearized and heat-denatured Bluescript plasmid), and incubated with hdDNA on ice for 10 min. Condensin or the SMC dimer was then added. The annealing reaction was terminated with loading dye in the absence of SDS followed by electrophoresis. For the RNA elimination assay, 830 nt RNA was made by *in vitro* transcription T7 (TaKaRa). See electronic supplementary material, figure S5.

### 5.8. Gel shift assay

Synthetic 86 nt ssDNA [59] labelled with fluorescein isothiocyanate (FITC; Sigma-Aldrich) was incubated with a series of concentrations of condensin, the SMC dimer or individual subunits in 20 µl of binding buffer (20 mM Tris–HCl at pH 7.5, 50 mM NaCl, 2 mM MgCl_2_, 10 per cent glycerol, 1 mM dithiothreitol) for 10 min at 30°C. The mixtures were then analysed in 10 per cent non-denaturing polyacrylamide gels made in 0.5 × TBE (44.5 mM Tris-borate, 1 mM EDTA) buffer, followed by fluorescent imaging (Typhoon9200). The same conditions were used for M13 ssDNA, but the samples were analysed in 0.7 per cent native agarose gels, followed by SYBR Gold staining (Molecular Probes). For RNA-binding assay, 4 per cent agarose (the NuSieve 3:1, TaKaRa) gel was used.

### 5.9. Atomic force microscopy imaging

AFM imaging was performed as previously described [4,21].
